# LncRNAs Involved in Antioxidant Response Regulation as Biomarkers of Gestational Diabetes: A Study on *H19*, *MALAT1* and *MEG3*

**DOI:** 10.3390/antiox13121503

**Published:** 2024-12-10

**Authors:** Jovana Stevanović, Uroš Petrović, Ana Penezić, Ognjen Radojičić, Daniela Ardalić, Milica Mandić, Vesna Mandić-Marković, Željko Miković, Miloš Brkušanin, Olgica Nedić, Zorana Dobrijević

**Affiliations:** 1 Institute for the Application of Nuclear Energy, University of Belgrade, 11080 Belgrade, Serbia; jovana.stevanovic@inep.co.rs (J.S.); anap@inep.co.rs (A.P.); olgica@inep.co.rs (O.N.); 2 University Clinic for Gynecology and Obstetrics “Narodni Front”, 11000 Belgrade, Serbiaardalic.daniela@gakfront.org (D.A.); vesna.mandic-markovic@med.bg.ac.rs (V.M.-M.); zeljko.mikovic@med.bg.ac.rs (Ž.M.); 3 Medical School, University of Belgrade, 11000 Belgrade, Serbia; 4 Centre for Human Molecular Genetics, Faculty of Biology, University of Belgrade, 11000 Belgrade, Serbia; milosb@bio.bg.ac.rs

**Keywords:** H19, MALAT1, MEG3, oxidative stress, redox status, inflammation, gestational diabetes, zinc, glutathione reductase, thiol content

## Abstract

Recent findings highlighted the potential of long non-coding RNAs (lncRNAs) as novel indicators of gestational diabetes mellitus (GDM), as they demonstrate altered expression in metabolic disorders, oxidative stress (OS) and inflammation (IFM). The aim of this study was to evaluate the diagnostic potential and prognostic significance of the OS/IFM-related lncRNAs H19, *MALAT1* and *MEG3* in GDM and their correlations with redox status-related parameters. The relative quantification of selected lncRNAs from peripheral blood mononuclear cells (PBMCs) of GDM patients and controls (n = 50 each) was performed by qPCR. The expression levels were tested for correlations with metal ion concentrations, NRF2 expression, activities of glutathione reductase (GR), superoxide dismutase (SOD), catalase (CAT), serum thiol content, protein carbonyl level and thiobarbituric acid reactive substances. *MALAT1* and *H19* were significantly downregulated in GDM patients (*p* = 0.0095 and *p* = 0.012, respectively). A correlation was observed between *H19* expression and zinc levels in both GDM patients and controls. *MALAT1* expression positively correlated with *NFE2L2* levels in GDM patients (*p* = 0.026), while *H19* exhibited a positive correlation with GR activity in controls (*p* = 0.018) and an inverse correlation with SOD activity (*p* = 0.048). Our data show the disturbance of OS/IFM-lncRNAs in GDM pathogenesis and illustrate the biomarker potential of the analyzed lncRNAs, as well as of certain redox status parameters.

## 1. Introduction

Gestational diabetes mellitus (GDM) is defined as a condition of carbohydrate intolerance of varying severity that either develops or is first identified during pregnancy [1]. GDM is one of the most prevalent medical complications of pregnancy, which is not surprising based on the rising incidence of obesity, metabolic disorders, undiagnosed hyperglycemia and even overt diabetes among young women of reproductive age [2]. Recent data from the International Diabetes Federation (IDF) published in 2021 reveal that the global standardized prevalence of GDM is 14.0% [3]. This high prevalence raises concern as hyperglycemia, coupled with elevated oxidative stress and inflammation, is associated with numerous complications during pregnancy and childbirth, which can have significant long-term effects on both maternal and offspring health. Beyond immediate childbirth complications, infants born by mothers with GDM face long-term health risks, including increased likelihood of obesity, hypertension, insulin resistance, type 2 diabetes (T2DM) and the associated cardiovascular issues [4].

A current method for diagnosing GDM involves an oral glucose tolerance test (OGTT) [5]. However, the test causes significant discomfort, nausea or even more severe health issues in pregnant women, for which reason it often requires repetition or may be contraindicated in women with severe glucose intolerance and non-diagnosed pre-gestational diabetes. There is also a lack of consensus on various aspects of the OGTT, such as the optimal glucose level cut-off values, test duration, sampling intervals and glucose quantity [6]. This highlights the need for improved indicators to better identify pregnant women at risk for GDM and to optimize their selection for OGTT testing. Since elevated glucose levels in earlier stages of pregnancy trigger mechanisms linked to negative outcomes associated with GDM, timely diagnosis and adequate intervention could significantly improve pregnancy outcomes [7]. Consequently, research efforts have intensified to identify biomarkers that can aid in diagnosing, predicting, or detecting obstetric and neonatal consequences of GDM.

Accumulating evidence linking metabolic dysregulation to non-coding RNAs has brought increased attention to this class of biomacromolecules, particularly among researchers investigating the molecular pathogenesis of GDM and their potential as biomarkers in liquid biopsy studies. Recent findings have highlighted the potential of long non-coding RNAs (lncRNAs) as novel indicators of GDM and the associated traits, as they exhibit regulatory functions in metabolic processes and demonstrate altered expression in metabolic disorders, oxidative stress and inflammation [8,9]. Through their participation in antioxidant response and inflammatory pathways, dysregulated lncRNAs may contribute to severe GDM-related complications, such as premature membrane rupture, disorders of amniotic fluid and intrauterine growth restriction. Furthermore, the altered expression of specific lncRNAs may be utilized for monitoring or predicting the onset of these pregnancy complications.

Peripheral blood mononuclear cells (PBMCs) have emerged as a promising source of diabetes-related non-coding RNAs and may represent a suitable sample for assessing the expression of GDM-related lncRNAs [10,11]. These cells play a pivotal role in immune response, with their functions being notably influenced by oxidative stress and interactions with free radicals. Additionally, as effector cells, PBMCs secrete cytokines and modulate inflammatory pathways, contributing to the chronic low-level inflammation accompanying hyperglycemia, pregnancy and GDM [12]. Encouraged by previous results that suggest non-coding RNAs derived from PBMCs may act as indicators of GDM and the metabolic status in pregnancy, we focused on PBMC-derived lncRNAs as novel GDM biomarker candidates in this study.

In this study, we focused on three lncRNAs: *MALAT1*, *MEG3* and *H19*, due to their previously reported roles in regulating glucose metabolism, insulin resistance, inflammation and antioxidant response. These regulatory characteristics make them potentially important players in the development and progression of GDM [13]. The lncRNAs *MALAT1*, *MEG3* and *H19* were previously reported as dysregulated in the circulation, placenta and umbilical cord blood of GDM patients and their offspring [14]. However, the expression of these lncRNAs in PBMCs has not yet been evaluated in the context of GDM diagnosis, prognosis and metabolic status evaluation. Furthermore, these lncRNAs are implicated in mechanisms underlying T2DM pathogenesis, with *MEG3* and *H19* originating from imprinted loci crucial for pregnancy maintenance, fetal growth and fetoplacental development [15].

Given the limited data on lncRNA expression in the PBMC of GDM patients, quantifying selected lncRNAs may help to identify novel biomarkers for GDM and clarify the role of lncRNAs in the metabolic dysregulation associated with GDM. By comparing the expression levels of *MEG3*, *MALAT1* and *H19* in PBMCs obtained from GDM patients and healthy pregnant women, we aimed at assessing their diagnostic potential. In addition, the present study aimed to evaluate the prognostic significance of the selected lncRNA pool. The results were analyzed in relation to the clinical data of the participants and their neonates to explore the potential links between the dysregulation of PBMC-derived lncRNA and the metabolic status of pregnant women. Furthermore, we examined several redox status-related parameters at the time of sampling (24–30 weeks), including indicators of lipid peroxidation, thiol content and protein carbonylation, as well as antioxidant enzyme activities and the expression levels of *NRF2*, a master regulator of antioxidant response. We evaluated GDM-related differences for these parameters, relation to the occurrence of adverse pregnancy outcomes, as well as possible correlations with the expression of selected lncRNAs.

## 2. Materials and Methods

A total of 50 patients diagnosed with GDM and 50 healthy normoglycemic pregnant women participated in the present study. Data and biological samples were collected at the Gynecology and Obstetrics Clinic “Narodni Front” (OGC NF), Belgrade, Serbia. This study was conducted in accordance with the principles outlined in the Helsinki Declaration from 1975 and was approved by the Ethics Committee of the Obstetrics and Gynecology Clinic “Narodni Front” (ethics committee approval no. 05006-2019-4925 dated 18 March 2019). Written informed consent was obtained from all participants, and relevant demographic, anthropometric and clinical data were recorded for each subject. Details of patient recruitment, selection criteria and database construction were previously described by Radojičić et al. [16]. Briefly, the following information was extracted from medical records: results of the diagnostic OGTT, other biochemical results monitoring glucose and lipid status, metabolism of redox-active metals, liver and kidney function, as well as biochemical and hematological indicators of inflammation and anemia. Information on pregnancy and delivery complications relevant to GDM, along with the anthropometric characteristics of the neonates, was also collected. Participants completed a tailored questionnaire to provide data for eligibility and correlation analyses, including age, height, self-reported weight (pre-pregnancy and at the time of sampling), gestational age, smoking status, number of childbirths and miscarriages, personal and family history of gestational or other type of diabetes and details of therapy and dosing regimen. Peripheral blood samples were collected from each pregnant woman in EDTA-containing tubes after overnight fasting, between the 24th and 30th week of pregnancy. The result of the OGTT test with 75 g of glucose was used to diagnose GDM following the criteria defined by the International Association of Diabetes and Pregnancy Study Groups (IADPSG). Only samples from participants without additional pregnancy pathologies or previously diagnosed metabolic disorders were included in subsequent analyses.

### 2.1. Isolation of Peripheral Blood Mononuclear Cells

The separation of PBMCs from whole peripheral blood samples was previously described in Radojičić et al. [16]. Buffy coats from blood centrifuged at 400× *g* (10 min at 4 °C) (Centrifuge 5804R, Eppendorf, Hamburg, Germany) were collected, diluted with phosphate-buffered saline (PBS, 10 mM, pH 7.4) and layered onto a lymphocyte separation medium (LSM 1.077 g/mL; Capricorn Scientific, Ebsdorfergrund, Germany). PBMC isolation was performed following the LSM manufacturer’s protocol, with minor adjustments: the separation of PBMCs from other white blood cells, left-over platelets and red blood cells was performed by centrifugation at 700× *g* for 30 min (at 18–21 °C); the centrifuge was set to the lowest acceleration, and the brake was disengaged; layered PBMCs were collected and washed twice with PBS by centrifugation at 100× *g* for 10 min (at room temperature). A final washing of the PBMC pellet was carried out using erythrocyte lysis buffer (155 mM NH_4_Cl, 10 mM KHCO_3_, 0.1 mM EDTA, pH 7.2) at 100× g for 10 min, after a 5 min incubation, at room temperature. The acquired cellular pellet was lysed in TRIzol reagent (Thermo Fisher Scientific, Waltham, MA, USA) and stored at −80 °C for subsequent RNA extraction.

### 2.2. Isolation and Quantification of Total RNA

The isolation of total RNA from PBMCs lysed in TRIzol was performed by phase separation and alcohol precipitation according to the protocol of the TRIzol reagent manufacturer, while the quantification and RNA purity assessment was performed spectrophotometrically, based on the absorbance at 260 and 280 nm (Epoch microplate spectrophotometer, BioTek Instruments–Agilent, Santa Clara, CA, USA).

In brief, phase separation was achieved by adding chloroform and centrifugation at 12,000× *g* for 15 min at 4 °C. The aqueous phase was collected, and RNA precipitation was performed using ice-cold isopropanol with the addition of glycogen. After 30 min of incubation at 4 °C, the mixture was centrifuged at 12,000× *g* for 10 min at 4 °C to obtain the RNA pellet. The pellet was washed with 75% ethanol and centrifuged at 7500× *g* for 5 min at 4 °C. After air-drying, the RNA was dissolved in DEPC-treated water by incubating at 56 °C on a thermoblock (Biosan TS-100C, Riga, Latvia). The extracted RNA was then stored at −80 °C.

### 2.3. Relative Quantification of lncRNAs and mRNAs

In the reverse transcription step, the reaction mixture contained 500 ng of RNA, 1.25 µM random hexamer primers (Thermo Fisher Scientific, Waltham, MA, USA), 2.5 µM oligo-dT primers (Thermo Fisher Scientific, Waltham, MA, USA), 0.5 mM dNTPs (each), 200 U RevertAid reverse transcriptase and 1× of the supplied RT buffer (Thermo Fisher Scientific, Waltham, MA, USA) with DEPC-treated water in a total volume of 20 µL. The protocol consisted of two phases:

Initially, RNA, DEPC-water, random hexamer primers and oligo-dT primers were combined and incubated at 70 °C for 5 min in a thermal cycler (Applied Biosystems 2720 Thermal Cycler, Thermo Fisher Scientific, Waltham, MA, USA) to denature the RNA, after which the mixture was immediately transferred to ice. Primer binding to RNA was facilitated during a 5 min incubation at 25 °C, followed by the addition of a mixture of reaction buffer, reverse transcriptase, and dNTPs. The reaction proceeded with incubation steps at 25 °C for 10 min, 42 °C for 60 min, and 72 °C for 10 min. Complementary DNA was stored at −20 °C until the relative quantification reaction.

The relative quantification of the lncRNAs *MALAT1* (NCBI Reference Sequence: NR_002819.4), *H19* (NCBI Reference Sequence: NR_002196.2), *MEG3* (NCBI Reference Sequence: NR_002766.2) and *NFE2L2* (NRF2, NCBI Reference Sequence: NM_006164.5) mRNA was performed by the qPCR method, with *GAPDH* (NCBI Reference Sequence: NM_002046.7) serving as the reference gene. Sequences of the specific primer pairs for each lncRNA and mRNA target are listed in Table 1, together with the corresponding references. The reaction was carried out according to the manufacturer’s recommendations for qPCR reagents (Thermo Fisher Scientific), whereby the reaction mixture contained 1x SYBR™ Green PCR Master Mix (Thermo Fisher Scientific, Waltham, MA, USA), milli-Q water and equimolar concentrations of forward and reverse primers (0.375 µM for *H19*, 0.3125 µM for *MALAT1*, 1.25 µM for *MEG3* and 0.625 µM for *GAPDH* and *NFE2L2*). The reaction temperature profile included enzyme activation and initial cDNA denaturation at 95 °C for 10 min, followed by 40 cycles of denaturation and primer annealing/elongation at 95 °C for 15 s and at 60 °C for 1 min, respectively. A melting curve analysis was performed by incrementally increasing the temperature from 60 °C to 95 °C. The qPCR reactions were carried out in a 96-well plate format using a QuantStudio 5 Real-Time qPCR system (Thermo Fisher Scientific, Waltham, MA, USA), and results were analyzed using QuantStudioTM Design & Analysis Software v1.5.2 (Thermo Fisher Scientific, Waltham, MA, USA).

### 2.4. Western Blot Analysis

NRF2 and GAPDH were detected by Western blot analysis. Proteins from PBMCs lysed in TRIzol were extracted using the optimized protocol described in Stevanović et al. [22], loaded onto 8% denaturing polyacrylamide gel and electroblotted onto a nitrocellulose membrane. Protein immunoblot analysis was performed using an anti-human NRF2 (1:1000) polyclonal antibody (Thermo Fisher Scientific, Waltham, MA, USA) and an anti-human GAPDH (1:10,000) monoclonal antibody (Cell Signaling, Danvers, MA, USA) as primary antibodies. Horseradish peroxidase-conjugated sheep anti-rabbit IgG (1:10,000) was used as the secondary antibody. Visualization was achieved by using ECL substrate (Thermo Fisher Scientific, Waltham, MA, USA) and exposing the membrane to an X-ray film. Densitometric analysis was conducted applying ChemiDoc MP Imaging System (Bio-Rad laboratories, Hercules, CA, USA) and Bio-Rad Image Lab Version 6.1 software.

### 2.5. Activity of Antioxidant Enzymes

The activities of antioxidant enzymes (glutathione reductase—GR, superoxide dismutase—SOD and catalase—CAT) were measured in hemolysates stored at −80 °C after being flash-frozen in liquid nitrogen. GR activity was measured spectrophotometrically (Epoch microplate spectrophotometer, BioTek Instruments—Agilent, Santa Clara, CA, USA), according to Glatzle et al. [23]. The assay is based on the ability of GR to catalyze the oxidation of NADPH to NADP^+^, followed by a change in the absorbance at 340 nm. Briefly, 60 µL of potassium phosphate buffer (KPB, 100 mM with 1 mM EDTA, pH 7.5), 10 µL of oxidized glutathione (GSSG, 2 mM), 10 µL of EDTA (0.5 M), 180 µL of H_2_O and 10 µL of NADPH (1 mM) were mixed, followed by the addition of 20 µL of the sample (diluted in potassium phosphate buffer 10 times). The assay was performed in 96-well plates, while the absorbance at 340 nm was monitored for 150 s. The GR activity in the samples corresponded to ΔA and was calculated by using the extinction coefficient of NADPH at 340 nm, the sample volume and the dilution factor. The specific activity of GR was expressed as nmol of NADP^+^ per minute per gram of hemoglobin [23].

SOD activity was measured spectrophotometrically (Epoch microplate spectrophotometer, BioTek Instruments—Agilent, Santa Clara, CA, USA) according to Beauchamp and Fridovich [24]. The assay utilizes photochemical events to generate O_2_^−^ and nitro blue tetrazolium (NBT) to detect this radical. SOD is able to inhibit the formation of the blue formazan dye and, thus, can be quantified. Briefly, 175 µL of KPB, 10 µL of methionine (13 µM), 8 µL of NBT (75 µM) and 6 µL of sample were mixed in a well of 96-well plate, followed by an addition of 1 µL of riboflavin (2 µM). Plates were incubated under UV light for 30 min, absorbance at 560 nm was measured upon incubation and ΔA was calculated (A_blank_ − A_sample_), representing the inhibitory activity of SOD. The IU of SOD activity was assessed as 50% of the inhibition. The specific activity of SOD in the analyzed samples was presented as IU/mg of hemoglobin [24].

CAT activity was assayed spectrophotometrically according to the method of Claiborn [25]. The principle of the assay is to monitor the decomposition of hydrogen peroxide (H_2_O_2_) catalyzed by CAT by measuring the absorbance at 240 nm for 60 s. Briefly, 202.5 µL of KPB and 22.5 µL of 50 mM H_2_O_2_ were mixed in a quartz cuvette, followed by the addition of 22.5 µL of the sample, and the absorbance at 240 nm was monitored immediately. The activity of CAT corresponded to ΔA and was calculated by using the extinction coefficient of H_2_O_2_ at 240 nm, the sample volume and a dilution factor. The specific activity of GR was expressed as μmol of the reduced H_2_O_2_ per minute per gram of hemoglobin [25]. All measurements were performed in triplicate.

### 2.6. Serum Thiol Content

Spectrophotometric analysis was used for measuring serum thiol content by Ellman’s method in a 96-well plate format. The assay uses chromogenic 5,5′–dithiobis(2-nitrobenzoic acid) (DTNB), which reacts with thiol groups, resulting in the production of 2-nitro-5-thiobenzoic acid (TNB), which can be quantified by measuring the absorbance at 412 nm. Samples (30 µL) were mixed with equal volumes of 2 mM DTNB and 1 M Tris buffer (pH 8.0), made up to 300 µL with water, and the absorbance at 412 nm was recorded after a 30 min incubation period against the sample blank (in triplicate). The content of the thiol group was calculated using the value of the extinction coefficient of the reagent [26].

### 2.7. Serum Protein Carbonyl Content

The degree of total protein carbonylation (PCO) in individual serum samples was examined by determining the concentration of PCO after protein derivatization using 2,4-dinitrophenylhydrazine (DNPH), according to the widely used method of Levin et al. [27]. Briefly, 250 µL of 10% trichloroacetic acid (TCA) was added to 500 µL of a serum sample diluted with distilled water to the concentration of 10 mg/mL protein. The mixture was centrifuged at 1500× *g* for 5 min, the supernatant was discarded and the precipitated proteins were incubated with 250 µL of 10 mM DNPH in 2 M HCl for 30 min at 25 °C, with thorough mixing. A blank sample was prepared for each serum using only 2 M HCl instead of the DNPH reagent. Proteins were precipitated with 500 µL TCA, centrifuged as previously and the precipitate was washed twice with 1 mL of a solution of ethanol–ethyl acetate in a ratio of 1:1, with vigorous mixing and centrifugation in between. The final precipitate was dissolved in 1.5 mL of 2% sodiumdodecylsulphate (SDS) prepared in 0.08 M phosphate buffer pH 8.0 containing 0.05% EDTA, by incubating tubes in a thermal block at 37 °C for 20 min. The absorbance of dissolved proteins was measured at 375 nm, corrected for the sample blank and the concentration of PCO was calculated using the extinction coefficient of 22,000 M^−1^ cm^−1^. The results were expressed as nmol PCO per mg of protein.

### 2.8. The Content of Thiobarbituric Acid Reactive Substances in Serum

Serum samples were assayed for thiobarbituric acid reactive substances (TBARSs), which serve as indicators of lipid peroxidation, by spectrophotometric analysis. First, 0.1 mL of each serum sample was mixed with 0.2 mL of TBARS reagent (15% TCA, 2% HCl, 0.375% 2-thiobarbituric acid) and heated in a boiling water bath for 15 min, following which the mixture was cooled and centrifuged at 3000× *g* for 10 min. The supernatant was used for the measurement of chromogen absorbance at 532 nm against a blank reference in a 96-well plate format. Products of lipid peroxidation were expressed as TBARS μmol/L and calculated using the value of the extinction coefficient of 156,000 M^−1^ cm^−1^.

### 2.9. Statistical Analysis

Statistical analyses of lncRNA and mRNA expression levels, along with clinical parameters, were conducted using RStudio (RStudio Team, 2024). The normality of continuous variables was assessed using the Kolmogorov–Smirnov test. The F test was used to check differences in variance between groups, while the two-tailed Student’s *t*-test was used to compare the results with a normal distribution. For data significantly deviating from the normal distribution, the Mann–Whitney U test was applied. Categorical variables were analyzed using the χ^2^ test. Associations between expression levels and clinical parameters were analyzed with linear regression, reporting Pearson’s correlation coefficient (r) and corresponding *p* values. Statistical significance was set at a *p* value < 0.05.

## 3. Results

Table 2 summarizes the basic characteristics of the study participants. No significant differences were observed between patients with GDM and controls regarding age, gestational age, neonatal characteristics or pregnancy/birth complications. As expected, participants with GDM exhibited a higher prevalence of a family history of GDM, elevated pre-pregnancy weight and BMI; however, weight gain up to the time of sampling was comparable between the two groups (*p* = 0.79). HDL cholesterol levels were lower in the GDM group. Additionally, GDM patients exhibited significantly higher levels of hemoglobin, fibrinogen and uric acid, along with increased hematocrit and reduced concentrations of albumin and zinc. No significant differences were detected for other metal ions analyzed in this study between the patients and controls (Table 2).

**Figure 1:**
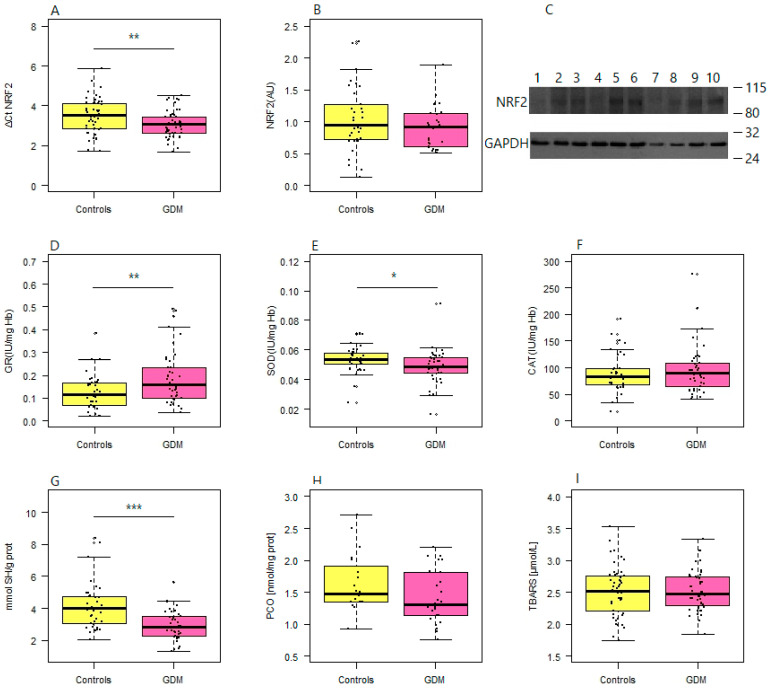
Differences in the redox status-related parameters between controls and GDM patients. (**A**) Relative expression of *NFE2L2* (encoding NRF2) in GDM vs. normoglycemic controls, presented as ΔCt values on the y-axis; (**B**) levels of the NRF2 protein in the GDM group and controls (**C**) with the representative immunoblot of samples belonging to GDM group (odd numbers) and controls (even numbers) paired according to the concentration of isolated proteins (on the right), demonstrating the lack of obvious differences in the expression of NRF2 between groups; specific activities of (**D**) GR, (**E**) SOD and (**F**) CAT in GDM patients and controls; (**G**) serum thiol, (**H**) PCO and (**I**) TBARS concentration in GDM patients and controls. Boxes represent medians with interquartile ranges, while whiskers indicate min and max values; statistical significance was analyzed by Student’s *t*-tests; *p* < 0.05 is indicated by an asterisk, *p* < 0.01 by two asterisks and *p* < 0.001 by three asterisks.

The results for other redox status-related parameters, the expression of *NFE2L2* and the specific activities of three antioxidative enzymes are displayed in Figure 1. The analysis of *NFE2L2* relative mRNA expression revealed a significant upregulation in the GDM group compared to the controls (*p* = 0.006) (Figure 1A). However, when converted to a fold change, the increase in the expression of this mRNA was merely 1.35 fold and there were no significant differences in the levels of NRF2 protein between the study groups (*p* = 0.262) (Figure 1B,C). The specific activity of GR was significantly higher in GDM patients than in healthy controls (*p* = 0.006) (Figure 1D). SOD activity (Figure 1E) and serum thiol content (Figure 1G) were both significantly reduced in the GDM group (*p* = 0.039 and *p* = 5.82 × 10^−5^). CAT activity, however, showed no significant difference between groups (*p* = 0.518) (Figure 1F). Serum PCO and TBARS concentrations were also similar in GDM patients and controls (*p* = 0.104 and *p* = 0.780, respectively) (Figure 1H,I).

**Figure 2:**
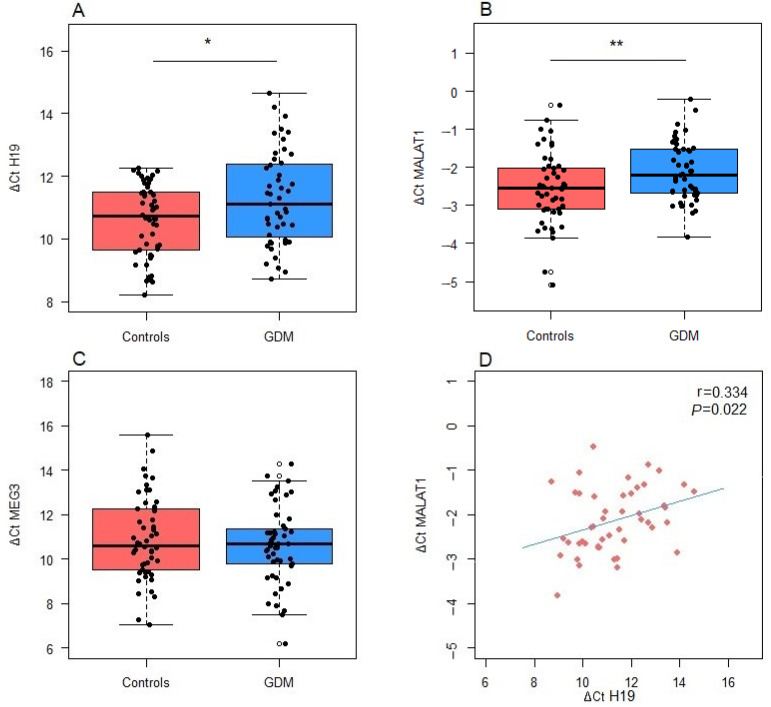
Differences in the expression levels of lncRNAs between GDM patients and controls: (**A**) *H19*, (**B**) *MALAT1* and (**C**) *MEG3*. (**D**) Correlation between the expression of *MALAT1* and *H19*. Boxes represent medians with interquartile ranges, while whiskers indicate min and max values; statistical significance was analyzed by Student’s *t*-tests; *p* < 0.05 is indicated by an asterisk and *p* < 0.01 by two asterisks. Pearson’s correlation coefficient ® and the corresponding *p* value from the linear regression analysis are presented within the scatter plot.

Figure 2 summarizes the results of lncRNA expression analyses. The expression of *MALAT1* and *H19* was significantly lower in GDM patients compared to healthy controls (*p* = 0.0095 and *p* = 0.012, respectively) (Figure 2A,B). In contrast, no significant differences in *MEG3* expression were observed between GDM patients and controls (*p* = 0.436) (Figure 2C). Furthermore, a positive correlation was determined between *MALAT1* and *H19* expression (r = 0.334, *p* = 0.022) (Figure 2D).

In examining correlations between the expression levels of analyzed lncRNAs and hematological/biochemical parameters in GDM patients, *H19* showed positive correlation with total protein and urea concentration. However, such correlations were absent in the control group (r = 0.324 and r = 0.335, respectively). For *MALAT1*, significant correlations were observed only within the control group, specifically with HDL concentration and leukocyte count (*p* = 0.034 and *p* = 0.016, respectively) (Table 3).

**Figure 3:**
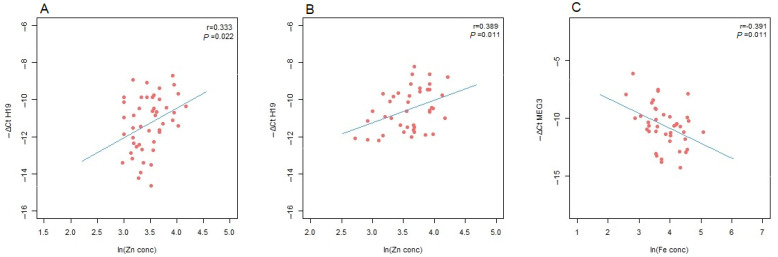
Correlations between the expression levels of lncRNAs and the logarithmically transformed values of metal ion concentrations: (**A**) *H19* and zinc in GDM patients; (**B**) *H19* and zinc in controls; (**C**) *MEG3* and iron in GDM patients. Pearson’s correlation coefficient (r) and the corresponding *p* value from the linear regression analysis are presented within the scatter plot.

In the analysis of metal ions, a statistically significant positive correlation was observed between H19 expression and plasma zinc levels in both GDM patients and controls (r = 0.333 with *p* = 0.022 and r = 0.389 with *p* = 0.011, respectively) (Table 3, Figure 3A,B). Conversely, a significant negative correlation was determined between MEG3 expression and iron concentration (r = 0.391, *p* = 0.011) (Table 3, Figure 3C).

**Figure 4:**
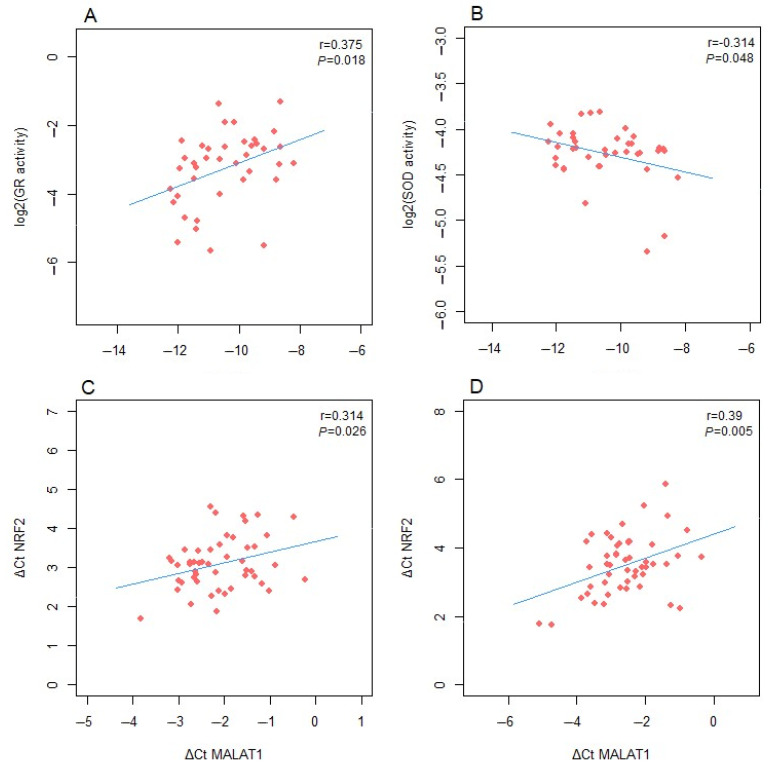
Correlations between the expression levels of lncRNAs (ΔCt) and the logarithmically transformed values of redox status parameters: (**A**) *H19* and GR activity; (**B**) *H19* and SOD activity; (**C**) *MALAT1* and *NFE2L2* mRNA expression in GDM patients; (**D**) *MALAT1* and *NFE2L2* mRNA expression in controls. Pearson’s correlation coefficient (r) and the corresponding *p* value from the linear regression analysis are presented within the scatter plot.

A positive correlation was found between *H19* expression and the specific activity of GR in controls (r = 0.375, *p* = 0.018) (Figure 4A). In contrast, an inverse correlation was observed between *H19* expression and the specific activity of SOD (r = 0.314, *p* = 0.048) (Figure 4B). A positive correlation was also noted between *MALAT1* expression and *NFE2L2* mRNA levels in GDM patients (r = 0.314, *p* = 0.026) (Figure 4C), as well as in controls (r = 0.390, *p* = 0.005) (Figure 4D).

**Figure 5:**
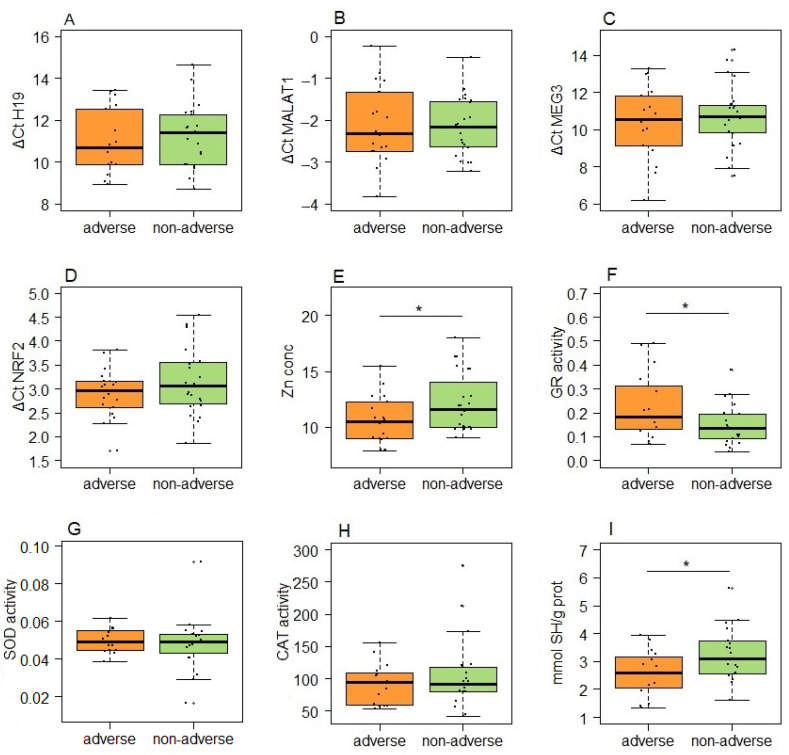
The expression of lncRNAs and redox status parameters in GDM patients stratified according to pregnancy outcomes: (**A**) *H19*; (**B**) *MALAT1*; (**C**) *MEG3*; (**D**) *NFE2L2* mRNA; (**E**) zinc concentration; (**F**) GR activity; (**G**) SOD activity; (**H**) CAT activity; (**I**) serum thiol content. Boxes represent medians with interquartile ranges, while whiskers indicate min and max values; statistically significant difference between two subgroups was analyzed by Student’s *t*-tests; *p* < 0.05 is indicated by an asterisk.

In our analysis of pregnancy outcomes in GDM patients, the lncRNAs selected for the analysis did not show a significant difference in expression between subgroups stratified by the occurrence of GDM-related obstetric/neonatal complications (Figure 5A–C). However, when comparing adverse versus non-adverse outcomes in GDM patients, several redox parameters demonstrated statistically significant differences. Specifically, levels of zinc, glutathione reductase (GR) activity and serum thiol content showed *p* values of 0.043, 0.042 and 0.045, respectively (Figure 5). Other parameters, including PCO and TBARS, which are not presented in Figure 5 (*p* = 0.601 and *p* = 0.434, respectively), remained insignificantly different in GDM patients with adverse pregnancy outcomes, compared to those without complications.

**Figure 6:**
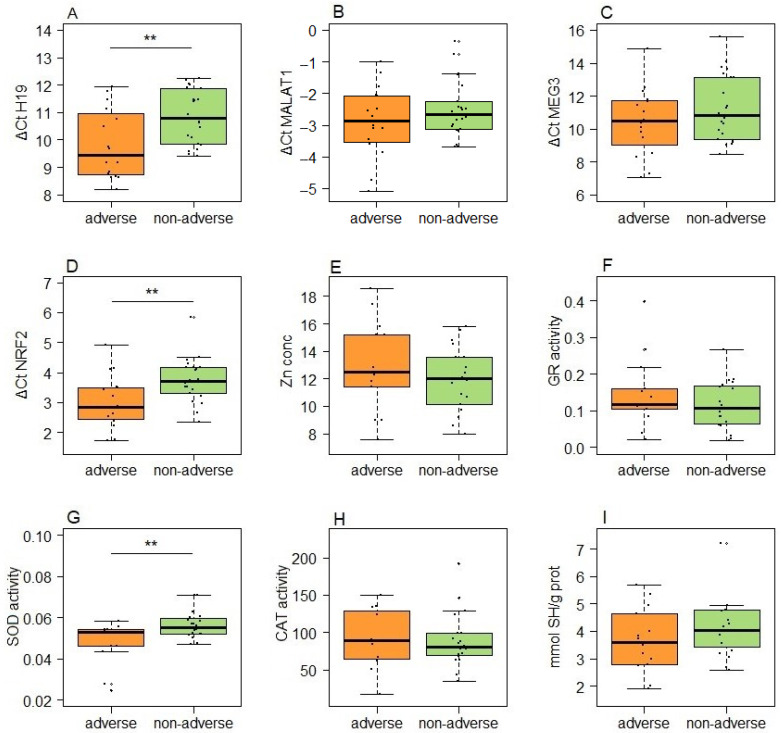
The expression of lncRNAs and redox status parameters in controls stratified according to pregnancy outcomes: (**A**) H19; (**B**) MALAT1; (**C**) MEG3; (**D**) NFE2L2 mRNA; (**E**) zinc concentration; (**F**) GR activity; (**G**) SOD activity; (**H**) CAT activity; (**I**) serum thiol content. Boxes represent medians with interquartile ranges, while whiskers indicate min and max values; statistically significant difference between two subgroups was analyzed by Student’s *t*-tests; *p* < 0.01 is indicated by two asterisks.

A different set of tested parameters proved to be predictive of adverse pregnancy outcomes in healthy women (Figure 6). Normoglycemic controls experiencing pregnancy complications and unfavorable outcomes exhibited a twofold increase in H19 expression (*p* = 0.008). Furthermore, there was a significant difference between subgroups regarding the expression of *NFE2L2* mRNA and SOD activity (*p* = 0.009 for both parameters).

## 4. Discussion

Due to its high prevalence, the lack of validated diagnostic and prognostic biomarkers and the association with severe obstetric and neonatal complications, the management of GDM was recognized as one of the leading problems in the clinical practice of obstetrics and reproductive endocrinology [28,29]. The associated risk of long-term disability resulting from GDM-related complications during pregnancy and at childbirth, the predisposition to T2DM and metabolic disorders in the offspring as well as the associated costs further support the significance of GDM as a pressing global health issue and a substantial burden on healthcare systems [29,30]. Consequently, there is an urgent need to enhance our understanding of the molecular pathogenesis of GDM and to identify clinically relevant biomarkers that could inform the development of diagnostic and prognostic algorithms. This necessity is further emphasized by the rising trends of obesity, sedentary lifestyles, modern dietary patterns and advanced maternal age [31,32].

Research into biomarkers for GDM has primarily focused on glycated forms of circulatory proteins, which are useful for monitoring glycemic status in other types of diabetes. However, glycated hemoglobin (HbA1c) and other plausible candidates thus far exhibit limited diagnostic significance, while the prognostic potential, in terms of adverse pregnancy outcomes, was poor or demonstrated low reproducibility [33,34]. More recently, ncRNAs have emerged as promising candidates in liquid biopsy approaches for the diagnosis, prediction and prognosis of GDM due to their known diverse regulatory roles, abundance in circulation and desirable chemical properties. Most of these studies analyzed microRNA molecules, while lncRNAs are gaining more attention, with technical advancements allowing their profiling and reliable quantification and with the accumulation of knowledge on their involvement in regulation of crucial cellular processes related to glucose and lipid metabolism, oxidative stress, inflammation and placentation [33,34,35]. Despite evidence suggesting that oxidative stress and a persistent low-grade inflammatory state may contribute to severe pregnancy complications associated with hyperglycemia [36,37], the specific changes in redox status related to GDM remain poorly understood. Therefore, besides analyzing the potential dysregulation of lncRNAs involved in the regulation of antioxidant response and inflammatory pathway in GDM, we aimed to thoroughly examine the changes in the activities of antioxidant enzymes in circulation and the levels of redox-active and inflammation-related metal ions, as well as serum thiol, TBARS and PCO concentrations in GDM, their correlations with lncRNA expression and their predictive significance.

In our present study, we focused on quantifying *H19*, *MALAT1* and *MEG3* in PBMCs, which are infrequently utilized as samples in research on liquid biopsy markers. However, using this type of easily-obtainable biological material as a source of ncRNAs with the promising biomarker properties in GDM proved justified in our previous study focused on microRNAs [16]. Furthermore, as this selected set of lncRNAs is involved in the regulation of antioxidant response and inflammatory pathways [38,39,40,41], PBMCs represent a cell type with expected dysregulation in their expression, since they are the effectors of the immune response, and they are directly exposed to redox-active species in circulation. To date, the majority of data on the role of these lncRNAs in GDM and other diabetic conditions during pregnancy have been acquired through studies analyzing the changes in their expression and their functional significance in placental tissues [14,42,43]. These studies demonstrated the involvement of *H19*, *MALAT*1 and *MEG3*, as well as lncRNAs in general, in the crucial processes required for maintaining a healthy pregnancy and in pathological processes associated with hyperglycemia. Since GDM is a systemic disease, we hypothesized that alterations in lncRNA expression would likely be reflected in other affected tissues, including blood cells representative of the inflammatory status in diabetic conditions, obesity and other metabolic disorders.

Our findings suggest that the expression of *H19* and *MALAT1* is significantly lower in PBMCs acquired from GDM patients, compared to normoglycemic controls. As for *H19*, our results are consistent with previous findings of its downregulation in the cord blood of neonates born from GDM pregnancies and in the placentas of GDM patients. This downregulation of *H19* in the cord blood and placental tissue in GDM has been associated with alterations in DNA methylation patterns, as demonstrated in GDM, and correlates with the level of hyperglycemia [14,44]. Furthermore, insulin resistance and glucose intolerance in male offspring born of GDM pregnancies have been linked to DNA hypermethylation-related downregulation of *H19*, as well as *IGF2*, which originate from the same imprinted locus [45]. The level of methylation of the *IGF2*/*H19* locus in GDM placentas was shown to correlate with the level of intrauterine hyperglycemia, as well as with the birth weight of the neonates [14].

In line with our current findings and previous reports on GDM, reduced *H19* expression has been observed in serum samples and serum-derived exosomes of patients with T2DM, as well as in T2DM skeletal muscles and in animal models with induced insulin resistance [46,47]. However, research on T2DM also found an upregulation of *H19* in plasma samples, making these previous T2DM-related results conflicting [48]. Even though there are contradictions regarding the direction of the established dysregulation of *H19* in diabetic states related to GDM, functional analyses across various tissue types clearly demonstrated its role in regulating glucose homeostasis, insulin signaling, glucose-induced inflammation and endothelial–mesenchymal transition in diabetic complications [47,49,50,51]. The main mechanisms through which this lncRNA acts in diabetes and pregnancy-related processes involve the regulation of activities of both microRNAs and enzymes involved in the epigenetic regulation of gene expression [47,49,50,51]. For instance, the suppression of *H19* has been shown to protect endothelial cells from high glucose-induced inflammation and oxidative stress by upregulating miR-29b, which plays a role in glucose balance and insulin secretion, impacting diabetes pathogenesis [52]. *H19* also targets other diabetes-related microRNAs, such as members of the let-7 family, miR-19b and miR-200b, through molecular sponging mechanisms [47,51,53]. Additionally, *H19* acts as a precursor of miR-675, shown to regulate trophoblast cell proliferation. Experimental data further support the role of *H19* in the epigenetic regulation of the imprinted locus *IGF2*/*H19*, affecting the expression of *IGF2*, a crucial metabolic regulator of glucose homeostasis and cellular growth [11,54,55].

*MALAT1*, the second lncRNA downregulated in PBMCs in our GDM group, has previously been reported as upregulated in the serum of GDM patients from China [56]. However, in this earlier study, patients with GDM were recruited in the later stage of pregnancy, which could significantly influence the results, due to potential variations in gene expression during the progression of pregnancy. *MALAT1* upregulation was also detected in placentas from GDM patients [42], which may not reflect the expression of *MALAT1* in previous stages of pregnancy, since all placentas were obtained at delivery. Similarly, studies on *MALAT1* expression in circulation in other diabetes types and associated cardiovascular complications present conflicting results [46,56]. The correlation between *MALAT1* and *H19* expression observed in our study could be a result of the presence of the same stimuli inhibiting the expression of these lncRNAs. A similar correlation was reported by Zhang et al. [13], which supports the validity of our finding.

Our results, suggesting a lack of significant change in the expression of *MEG3* in GDM, are inconsistent with previous findings, which demonstrated the downregulation or upregulation of this lncRNA in the placentas of mothers with GDM [43,57]. *MEG3* also showed dysregulated expression in different tissue types of patients with other diabetic conditions [58]. However, *MEG3* expression in PBMCs in the context of GDM is less likely to correlate with expression in the placenta compared to other candidate lncRNAs, since the placenta is among tissue types that exhibit the highest *MEG3* levels, while PBMCs show markedly lower expression.

The lncRNAs tested in this study did not demonstrate a strong potential as biomarkers of metabolic status in our cohort of pregnant women. However, a positive correlation was found between the expression level of *H19* and serum zinc level in GDM patients and controls. Both *H19* and serum zinc levels are reduced in GDM, which is consistent with the determined correlation. While zinc levels were predictive of adverse pregnancy outcomes in GDM [59], this predictive capacity was not observed for *H19*. Although we did not detect a significant change in the expression of *MEG3* between GDM patients and controls, our results demonstrated a moderate correlation between *MEG3* expression and serum iron levels in GDM. Therefore, *H19* and *MEG3* might serve as indicators of the status of these essential micronutrients in GDM or in pregnancy in general. Taking into account the effect of maternal nutrition on the methylation of imprinted loci [60,61], such as the ones from which both of these lncRNAs originate from, the established correlations are not surprising.

Since a significant difference in the activity of GR in blood cells, serum thiol content and the expression of NRF2 mRNA between GDM patients and controls was detected, the potential correlations of the expression levels of lncRNA with the values of redox status-related parameters were examined. The most significant finding was an inverse correlation of *MALAT1* expression with the expression of NRF2 mRNA in both GDM patients and controls. Previous evidence supports the inhibitory effect of *MALAT1* on NRF2 expression [62,63]. However, this lncRNA also exhibited negative effect on KEAP1 expression, which is a partner protein and an endogenous inhibitor of NRF2 [64,65], suggesting a complex, context-dependent relationship between *MALAT1* and NRF2. Other lncRNAs showed no evidence of correlations with redox status indicators in GDM patients. However, in healthy normoglycemic controls, *H19* expression negatively correlated with SOD activity and positively with GR activity. These correlations could be relevant for the prediction of the occurrence of adverse outcomes in normoglycemic pregnancies, since both *H19* expression and SOD activity differed between controls with later pregnancy complication and those with uncomplicated pregnancies. In GDM patients, however, GR activity and serum thiol content were better predictors of pregnancy outcome than lncRNAs. Therefore, in this cohort of GDM patients, the analyzed lncRNAs did not show a predictive value for adverse pregnancy outcomes.

Analysis of the pre-pregnancy body weight and BMI between GDM patients and controls showed that there was a significant difference between the two groups, which might have influenced the results due to the high impact of obesity on redox status and IFM [66]. The observed difference is a consequence of the recruitment method and is expected, since increased body mass is a risk factor for GDM. However, at the sampling stage, differences in body weight between cases and controls were not statistically significant and we found no correlations between body weight, BMI or weight gain and lncRNA expression levels. Furthermore, these anthropometric characteristics did not show variations related to pregnancy outcomes in GDM patients and controls.

The results of our present pilot study provide the first evidence of the diagnostic significance of the lncRNAs *H19* and *MALAT1* from PBMCs in GDM. Furthermore, this is the first study to evaluate the biomarker properties of the selected lncRNAs isolated from PBMCs in GDM. We demonstrated the alterations in certain redox status-related parameters in GDM vs. normoglycemic pregnancy, as well as the prognostic significance of these parameters in terms of adverse pregnancy outcomes. Importantly, we observed correlations between the expression levels of selected lncRNAs and redox parameters, as well as with serum concentrations of redox-active or inflammation-related metal ions. However, this study has limitations, including a small sample size and a candidate-based approach that restricted our analysis to a limited set of lncRNAs. It should be noticed that this is a pilot study aimed at testing the plausibility of including (g)OS/IFM-related lncRNAs in GDM biomarker analyses, so the limited cohort size reduced the statistical power and might have influenced the significance of the obtained results. Some of the true associations may be missed, while the interpretation of the acquired statistically significant differences and correlations should be taken with caution. Therefore, validating these results in a much larger dataset through a prospective study is essential, while an increase in study power would improve the generalizability of the results and lead to an improved characterization of the relation between redox status-related parameters and lncRNA expression. A multicentric approach in the recruitment of patients, their monitoring and GDM management would be the most reasonable option for reaching the required sample size, while maintaining the required quality of the recruitment procedure, sampling, data processing and follow-up. Although *H19*, *MEG3* and *MALAT1* were selected merely as representatives of (g)OS/IFM-related lncRNAs for the pilot study in order to test the hypothesized relevance of this class of molecules as GDM biomarkers, we acknowledge the limitations of using candidates instead of lncRNA profiling. Broadening the analysis to other lncRNAs known to be involved in (g)OS/IFM mechanisms would certainly lead to a better understanding of the role of lncRNAs in GDM’s onset and the development of GDM-related complications. Furthermore, additional potential biomarkers of GDM could be identified in a larger pool of (g)OS/IFM-related lncRNAs. A follow-up and resampling at several time points would be valuable to assess potential gestational stage-related variations in the values of redox status parameters and in lncRNA expression. Namely, our study design was based on the evaluation of biochemical and molecular parameters during a specific range of pregnancy age. Multiple sampling points would allow for testing the role of the analyzed lncRNAs as early predictors of GDM and would be essential for assessing the robustness of the potential biomarkers, as well as for defining the adequate time-frame for the analysis. A statistically highly relevant and unbiased assessment of the relation between redox status-related changes and pregnancy outcome aimed at validating our findings requires a large group of carefully monitored and well-characterized GDM patients. Another limitation of the present study, which is the lack of data on dietary intake of energy and nutrients, can be overcome in a larger study which would test the effects of nutrition on pregnancy outcome and provide meaningful estimations of its confounding effect. Despite these limitations, our data support the relevance of both g(OS)/IFM and the related lncRNAs for GDM development, monitoring and prognosis of adverse effects and illustrate the biomarker potential of analyzed lncRNAs and certain redox status parameters.

## 5. Conclusions

According to our findings, some of the parameters of antioxidant response in mid-pregnancy show significant differences between patients with GDM and healthy pregnant controls. Furthermore, these parameters demonstrate a discriminatory potential in terms of pregnancy outcomes. As for the principal molecules in focus in this study, i.e., the analyzed (g)OS/IFM-related lncRNAs, PBMC-derived *H19* and *MALAT1* exhibit the potential to serve as diagnostic biomarkers of GDM. These lncRNAs further show correlations with serum levels of metal ions, as well as with the activities of antioxidant enzymes and *NFE2L2* expression. However, these initial results require validation in a much larger prospective study, which should incorporate additional information on other redox status-related parameters, inflammatory mediators and nutritional factors, in order to define the exact (g)OS/IFM pattern of GDM pregnancies and the associated complications. Encouraged by the results of this relatively small pilot study, we propose the inclusion of other (g)OS/IFM-related non-coding RNAs in future assessments of novel GDM biomarkers.

## Figures and Tables

**Table 1:** Primer sequences and the corresponding references.

Primer	Sequence	References
GAPDH fw	5′-GAAGGTGAAGGTCGGAGT-3′	[17]
GAPDH rv	5′-GAAGATGGTGATGGGATTTC-3′	
H19 fw	5′-TGCTGCACTTTACAACCACTG-3′	[18]
H19 rv	5′-ATGGTGTCTTTGATGTTGGGC-3′	
MEG3 fw	5′-CCTCACCTCCAATTTCCTCTTC-3′	[19]
MEG3 rv	5′-CTTCCATCCGCAGTTCTTCA-3′	
MALAT1 fw	5′-GGTAACGATGGTGTCGAGGTC-3′	[20]
MALAT1 rv	5′-CCAGCATTACAGTTCTTGAACATG-3′	
NRF2 fw	5′-AGTGGATCTGCCAACTACTC-3′	[21]
NRF2 rv	5′-CATCTACAAACGGGAATGTCTG-3′	

**Table 2:** Basic characteristics of study participants.

	GDM Patients	Controls	*p* Value
N	50	50	
Age (years) ^a^	35.2 ± 4.2	33.4 ± 4.7	0.051
Gestational age at sampling (weeks) ^a^	27.2 ± 2.1	26.5 ± 1.6	0.056
Smoking status (%)	20	24	0.23
Family history of diabetes (%)	38	20	0.047 *
Pre-pregnancy weight (kg) ^a^	70.8 ± 13.0	66.4 ± 8.1	**0.049**
Weight at sampling (kg) ^a^	77.8 ± 12.2	74.4 ± 9.2	0.13
Height (cm) ^a^	169.0 ± 5.1	170.8 ± 6.6	0.14
Weight gain (kg) ^a^	6.8 ± 5.0	6.3 ± 11.3	0.79
Pre-pregnancy BMI	24.8 ± 4.7	22.8 ± 2.5	**0.009**
*Glycemic status* ^a^			
OGTT (mmol/L)			
0′	4.89 ± 0.66	4.42 ± 0.40	**4.24 × 10^−5^**
60′	10.84 ± 1.49	7.73 ± 1.21	**3.23 × 10^−19^**
120′	9.25 ± 1.93	6.6 ± 0.98	**4.15 × 10^−12^**
Fasting insulin (mU/L)	18.07 ± 15.85	18.67 ± 20.34	0.56
HOMA-IR	4.29 ± 4.71	3.26 ± 2.38	0.16
HbA1c (%)	4.78 ± 0.22	4.67 ± 0.23	0.09
*Lipid profile* ^a^			
Triglycerides (TGs) (mmol/L)	2.38 ± 0.79	2.15 ± 0.72	0.20
Cholesterol (mmol/L)	6.72 ± 1.14	6.86 ± 1.21	0.60
HDL (mmol/L)	1.85 ± 0.41	2.17 ± 0.50	**0.001**
LDL (mmol/L)	3.82 ± 1.03	3.71 ± 1.14	0.66
*Other biochemical parameters* ^a^			
Total proteins (g/L)	64.12 ± 3.42	64.31 ± 4.67	0.83
Albumin (g/L)	34.50 ± 2.37	35.97 ± 2.98	**0.021**
Urea (mmol/L)	3.17 ± 1.02	2.98 ± 0.78	0.35
Creatinine (µmol/L)	50.81 ± 7.09	51.80 ± 6.75	0.52
Uric acid (µmol/L)	239.76 ± 52.48	215.20 ± 38.16	**0.019**
CRP (mg/L)	6.80 ± 8.45	6.06 ± 4.24	0.61
AST (U/L)	17.50 ± 6.46	18.15 ± 5.26	0.67
ALT (U/L)	19.83 ± 10.47	16.06 ± 8.75	0.06
Bilirubin (µmol/L)	2.38 ± 0.57	2.80 ± 1.49	0.10
Fibrinogen (g/L)	3.88 ± 0.88	3.40 ± 0.71	**0.047**
Copper (µmol/L)	25.79 ± 8.81	24.56 ± 7.82	0.49
Zinc (µmol/L)	11.27 ± 2.44	12.45 ± 2.94	**0.042**
Iron (µmol/L)	15.36 ± 5.85	16.51 ± 8.04	0.48
Ferritin (µg/L)	20.48 ± 14.34	16.49 ± 13.06	0.11
TIBC (µmol/L)	59.96 ± 11.30	60.07 ± 10.08	0.96
Transferrin (g/L)	2.90 ± 0.31	2.92 ± 0.25	0.78
*Complete blood count* ^a^			
Erythrocytes (1012 cells/L)	3.80 ± 0.30	3.69 ± 0.36	0.14
Hemoglobin (g/L)	116.40 ± 7.95	110.25 ± 9.04	**0.0018**
Hematocrit	0.344 ± 0.025	0.329 ± 0.027	**0.012**
Sedimentation rate (mm/h)	34.55 ± 15.77	33.38 ± 12.50	0.72
Leucocytes (109 cells/L)	9.75 ± 2.15	9.62 ± 1.94	0.78
Thrombocytes (109 cells/L)	239.93 ± 60.82	237.42 ± 45.95	0.84
Granulocytes (109 cells/L)	6.98 ± 1.84	6.30 ± 2.20	0.14
Lymphocytes (109 cells/L)	2.11 ± 0.41	3.14 ± 3.77	0.51
*Neonates’ characteristics and obstetric complications* ^a^			
Weight (g)	3473.5 ± 480.3	3602.3 ± 457.5	0.26
Length (cm)	51.20 ± 2.44	51.39 ± 4.04	0.79
BMI	12.90 ± 0.95	13.15 ± 1.00	0.30
Apgar score at 1 min	8.88 ± 0.50	8.68 ± 1.00	0.28
Preterm labor (%)	17	7.9	0.22
Macrosomia (%)	14.6	18.4	0.65
Polyhydramnios (%)	9.8	7.9	0.77

^a^ Mean ± SD. * Statistically significant results are shown in bold. Abbreviations: GDM—gestational diabetes mellitus; BMI—body mass index; OGTT—oral glucose tolerance test; HOMA-IR—homeostatic model assessment of insulin resistance; HbA1c—glycated hemoglobin; TGs—triglycerides; HDL—high-density lipoprotein; LDL—low-density lipoprotein; CRP—C-reactive protein; AST—aspartate aminotransferase; ALT—alanine aminotransferase; TIBC—total iron-binding capacity.

**Table 3:** Correlations of the expression of *H19*, *MALAT1* and *MEG3* (−ΔCt) with the values (log_2_n) of the clinical parameters of GDM patients and healthy controls.

Group of Participants		*H19*		*MALAT1*		*MEG3*	
		r	*p* Value	r	*p* Value	r	*p* Value
**GDM patients**	*Lipid profile*						
	Triglycerides	−0.304	0.053	−0.219	0.17	−0.094	0.56
	Cholesterol	0.006	0.97	−0.150	0.35	−0.243	0.12
	HDL	0.192	0.18	0.027	0.85	0.217	0.13
	LDL	0.139	0.39	−0.074	0.64	−0.162	0.31
	*Glycemic profile*						
	Fasting glucose	−0.216	0.13	−0.108	0.45	−0.034	0.81
	OGTT 60′	−0.229	0.11	0.092	0.52	0.074	0.61
	OGTT 120′	−0.007	0.96	0.069	0.63	−0.235	0.10
	Fasting insulin	−0.059	0.68	−0.055	0.70	−0.018	0.90
	HOMA-IR	−0.116	0.45	−0.095	0.53	0.126	0.41
	HbA1c	−0.118	0.55	0.144	0.46	0.192	0.33
	*Other biochemical parameters*						
	Total proteins	**0.324**	**0.036**	0.049	0.76	0.167	0.29
	Albumin	−0.002	0.99	−0.199	0.22	−0.067	0.68
	Urea	**0.335**	**0.03**	0.062	0.70	−0.061	0.70
	Creatinine	0.018	0.91	−0.274	0.08	−0.245	0.12
	CRP	0.228	0.15	−0.169	0.29	0.037	0.82
	Fibrinogen	−0.129	0.45	−0.294	0.08	0.203	0.23
	Iron	−0.062	0.70	−0.018	0.91	**−0.391**	**0.01**
	Copper	−0.019	0.90	0.014	0.92	0.275	0.053
	Zinc	**0.333**	**0.018**	0.127	0.38	−0.164	0.26
	*Complete blood count*						
	Erythrocyte count	−0.006	0.97	−0.178	0.26	−0.068	0.67
	Hemoglobin	0.084	0.60	0.126	0.43	−0.089	0.58
	Hematocrit	−0.102	0.52	−0.156	0.32	−0.147	0.35
	Leukocytes	−0.302	0.052	−0.158	0.32	−0.022	0.89
	Thrombocytes	0.012	0.94	−0.076	0.63	0.189	0.23
	*Neonates’ characteristics*						
	Birth weight	0.135	0.45	0.102	0.56	0.052	0.77
	BMI	−0.081	0.65	−0.045	0.80	0.220	0.21
**Controls**	*Lipid profile*						
	Triglycerides	−0.065	0.70	0.198	0.23	−0.014	0.93
	Cholesterol	0.039	0.82	−0.123	0.46	0.007	0.97
	HDL	0.241	0.12	**−0.328**	**0.034**	0.206	0.19
	LDL	0.031	0.87	0.005	0.98	−0.202	0.28
	*Glycemic profile*						
	Fasting glucose	−0.010	0.94	0.034	0.81	0.142	0.32
	OGTT 60′	−0.080	0.58	0.063	0.66	−0.181	0.21
	OGTT 120′	0.197	0.17	−0.001	0.99	−0.002	0.99
	Fasting insulin	−0.152	0.34	−0.249	0.11	−0.046	0.77
	HOMA-IR	−0.115	0.51	−0.299	0.08	0.130	0.46
	HbA1c	0.140	0.46	−0.020	0.92	−0.042	0.82
	*Other biochemical parameters*						
	Total proteins	0.065	0.68	0.064	0.69	0.070	0.66
	Albumin	−0.180	0.30	0.106	0.54	**−0.334**	**0.049**
	Urea	0.053	0.74	−0.131	0.41	−0.001	0.99
	Creatinine	0.009	0.96	0.110	0.50	−0.062	0.70
	CRP	0.104	0.51	0.221	0.15	0.048	0.76
	Fibrinogen	0.027	0.91	0.380	0.11	0.050	0.84
	Iron	−0.034	0.85	−0.033	0.86	−0.016	0.93
	Copper	−0.228	0.15	0.045	0.78	−0.099	0.53
	Zinc	**0.389**	**0.012**	0.064	0.69	0.122	0.45
	*Complete blood count*						
	Erythrocyte count	0.259	0.11	0.219	0.17	−0.034	0.84
	Hemoglobin	0.282	0.08	0.260	0.10	0.052	0.75
	Hematocrit	0.169	0.30	0.295	0.06	−0.111	0.50
	Leukocytes	−0.210	0.19	**−0.379**	**0.016**	−0.248	0.12
	Thrombocytes	−0.135	0.41	−0.145	0.37	0.206	0.20
	*Neonates’ characteristics*						
	Birth weight	0.132	0.50	0.079	0.69	0.132	0.50
	BMI	0.164	0.40	0.201	0.30	0.166	0.40

Statistically significant results are shown in bold. Abbreviations: GDM—gestational diabetes mellitus; BMI—body mass index; OGTT—oral glucose tolerance test; HOMA-IR—homeostatic model assessment of insulin resistance; HbA1c—glycated hemoglobin; HDL—high-density lipoprotein; LDL—low-density lipoprotein; CRP—C-reactive protein; r—Pearson’s correlation coefficient.

## Data Availability

The raw data supporting the conclusions of this article will be made available by the authors on reasonable request.
